# The Annual Meeting of the Kentucky Dental Association

**Published:** 1873-08

**Authors:** 


### Proceedings of Societies.

### THE ANNUAL MEETING OF THE KENTUCKY DENTAL ASSOCIATION,

*In connection with the Indiana State Dental Association, was held June 5th. and 6th., at Louisville, Ky.*

### MORNING SESSION.

The joint meeting of the State Dental associations of Kentucky and Indiana, was called to order at half-past ten o'clock.

After prayer by Rev. J. H. Heywood, the president of the Kentucky Dental Association, W. H. Goddard, welcomed the Indiana association in the following words :

### WELCOMING ADDRESS.

*Mr. President, and Gentlemen of the Indiana State Dental Association:*

It becomes my official duty to extend to you, in behalf of the Kentucky State Dental Association, a welcome to our State, our city, and our fraternal hospitalities; and I fulfill the duty with all my heart; for in this case, official duty becomes th highest personal privilege. Yes—I regard it as my personal good that I am the organ of our Association in offices friendship and courtesy which enlist the warmest sympathies of which I am capable.

Accept then, Mr. President, for yourself and associates, the welcome which I extend to you; and believe me, the cordial words of greeting which I utter are truthful exponents of our feelings. Our hearts are on our lips, and in the grasp of our hands. Welcome among us—thrice welcome! Welcome to our homes, our fellowship, our hearts! We thank you for the opportunity you have given us of evincing our fraternal regards; and we trust that when you leave us to return to your homes, it will be with the bands of amity and friendship between our two societies more strongly cemented than before, and with personal ties renewed or freshly created which shall not be sundered this side of the grave!

By the programme of arrangements for our order of business, we are to join together as one body for discussions and interchange of thought and experience, and to that interesting feature of the occasion, let me devote a few passing words.

I am irresistibly led to notice the delightful contrast which the circumstances of this occasion present to the spirit, which in by-gone years marked the prevailing attitude of our professional brethren, each to the other For it is but a few years since each one of us considered it his duty to himself to look with suspicion on every professional brother, to conceal every new thought, every new discovery, closely within his own breast, his or own labratory; yes, even, perhaps to lock his door against the entrance of a brother dentist, who might perhaps, if admitted to confidence and intercourse, pillage some of the methods on which his host prided himself, and use them in his own operations.

There was no fraternity of feeling, no reciprocity of good offices, no free interchange of suggestion and experience. But we have come to feel, thank God, that all was contemptible by-play, unworthy of men of thorough scientific knowledge and progress. We have come to believe that when any man throws his contributions of thought, experience and skill into the common stock, he grandly benefits himself, as well as the world at large. This magnanimity and philanthropy return home to bless him more abundantly. Fraternity is now the word!

Knowledge loves to have the votaries who sit reverently at her feet clasp each other's hands in a close embrace as they do so, for her sake, for the world's sake, and for their own sakes! By as much as we claim to be lovers of truth and loversof science, above the petty arts of the selfish and narrow pretender, we glory in the inter-communication of knowledge and good offices. Now all are brothers; we are affiliated in associations; we meet to impart all we know to each other, and to put each other, if possible, on a higher plane of power and possibility. We do not fear our instruments, our materials, or our knowledge, will be stolen from us. On the contrary, our offices, and laboratories, with all they contain are open to the examination of every worthy brother. Yes, and our hearts and purses are open too.

It is interesting to note that this is only the second instance of the union of two state societies in joint sessions for mutual discussion and improvement; that of the Illinois and Iowa associations, which lately met together, having been the first. This is the second; may it not be the last! But may the exercises of this occasion so bind us together that we shall be eager for a repetition of the genial and profitable fraternity, and relations be this day inaugurated which shall be renewed with ever increasing luster and profit, so that in the far future our successors, when we are in our graves, shall rise up and call us blessed! Once more, Mr. President, I tend you a most cordial welcome.

In response, the president of the Indiana Dental Association, Dr. Wells, returned thanks in the name of the members of the association which he represented, to the Kentucky Association for the kindness and fraternal feeling which has been shown toward them, which they would not fail to reciprocate when the occasion offered. They had come to seek knowledge and light with their Kentucky brethren. Although separated by geographical lines, they were united in bonds of fraternity and affection, and he hoped they would continue ever to be so.

Dr. Priest read the minutes of the previous meeting, which were approved, when the following subject, the first on the programme, was announced as in order:

"Contour Filling—What are its advantages, what its permanency, and what the essential points to be observed?"

Dr. P. G. C. Hunt, of Indianapolis, who was selected by the Executive Committee to open the discussion on this point, arose and stated that he had with him a few casts of cases which had come under his treatment. He would prefer to change the subject a little, and would take his starting point at the last clause, namely: "What are the essential points to be observed?" The main subject under consideration was contour filling, or restoring a tooth to shape. He treated at some length of his methods of proceeding, saying that when a tooth is broken down, in whole or in part, the dentist must draw largely on his ingenuity. He presented casts of cases in which the teeth had been largely broken down, the teeth as restored often appearing to be principally constructed of gold. It is necessary to first remove all diseased parts, or all decayed bone, so as to have a healthful border for the filling. The next step is to secure the gold that there is no danger of its becoming loose. Screws he regarded as very valuable in some cases to secure contour filling, but he does not regard them as absolutely necessary in any case. He believed in letting the lateral enamel stand for ateral support for the filling. In many cases he fastened the filling by inserting gold wire in the root, an arrangement which he regards as more cleanly and more durable than the pivot tooth.

The advantages of contour filling are that it presents a smooth surface to the tongue, a better surface for mastication and for articulation.

In regard to permanency he had not much experience. He had been using the contour filling for three or four years, and found that it would wear, but not so rapidly as the two surfaces of bone would.

Dr. Hunt continued his subject at some length, describing processes and material. The discussion which followed took a wide range; over all the ground covered by the question as stated.

On motion the association agreed to meet at 9 o'clock, and adjourn at half-past 12 o'clock in the morning, and to meet at 3 o'clock and adjourn at half-past 6 in the evening.

After further discussion of the question presented for the morning session, the association adjourned at half-past 12.

### AFTERNOON SESSION.

The associations were called to order by the President of the Kentucky Association, Dr. W. H. Goddard, at twenty minutes after two o'clock.

The consideration of the second subject was passed over, as no member had any remarks to offer.

Dr. Taft of Cincinnati Dental College, read the following paper on the subject of diagnosis in practice, by Dr. D. C Hauxhwrst: (For which, see June REGISTER.—Ed.)

On this subject Dr. Taft remarked, that he considered it a very important one, relating to the daily practice of every den tist. Teeth are affected by causes remote from the mouth. These cases it is difficult to treat without knowing these remote causes. Disorders of other organs will extend their influence to the pulps of the teeth, whether exposed or not. In some cases of uterine irritation affecting the teeth, it is difficult to trace the action or to understand the cause. This action Dr. Taft illustrated with various cases which he has treated. Other nervous affections will extend their influence to the teeth and the parts around them. Dentists should understand wel he pathology of the system. It should be a subject of study with them; otherwise they are laboring in the dark. Considering the ignorance of some dentists on this point it is no wonder that their best operations often fail.

Dr. Taft continued on this subject for some time, endeavoring to show the necessity to the dentist of thorough knowledge of the human system.

Considerable discussion of the subject followed, and many interesting instances were adduced of cases bearing on this point.

The hext question was taken up for discussion.

"Importance of conserving the vitality of the teeth, and the best means of securing it."

Dr Taft lead the discussion on this point, a discussion which was participated in by most of the members.

The chair announced the following operators for the clinics of the following morning: Drs. Keely, S. B. Brown and Merritt Wells, of Indiana.

Also, that at half-past 4 o'clock the next day, Dr. Seymour would fill a tooth at his office, on Chesnut street, between Second and Third, by request.

Dr. W. F. Morrill then read the following paper: (For which see June REGISTER.—ED.)

It was moved that a publication Committee, consisting of three members—two from Kentucky and one from Indiana—be appointed. The motion was carried, and the President announced that he would name the committee the next day.

The joint session then rose, and the Kentucky association went into an election of officers, the following ticket being elected:

G. W. Priest, President.

W. M. Rogers, Vice President.

B. Oscar Doyle, Secretary.

J. F. Canine, Treasurer.

Board of Censors—Dr. Goddard, vice Dr. Redman, term expired.

Executive Committee—Drs. Priest, Canine, and Dunn, re-elected.

Dr. Canine extended an invitation to the members of the Associations, to attend a social "bee" at his house.

Following which the meeting adjourned.

JUNE 6th. MORNING SESSION.

The State Dental Association of Kentucky met at nine o'clock in the Hall of the School Board, at the corner of Walnut and Center streets, President Goddard presiding.

An amendment to the constitution, introduced by Dr. Doyle on Tuesday, was taken up for consideration, and its further consideration postponed until afternoon.

The President announced that he would appoint on the Publication Committee, the following gentlemen: Drs. W. F. Morrell, of New Albany; B. Oscar Doyle, and G. W. Priest. By virtue of the constitution, Dr. Doyle is chairman of this committee.

Dr. W. M. Rogers introduced the following amendment to article 6 of the constitution.

*Resolved*, That Article 6 of the Constitution of the Kentucky State Dental Association be so amended as to read: "The regular annual meetings of the Associations shall be held at such places as the Association from time to time may select."

This amendment was warmly discussed. The president ruled that the amendment must lie over one day, when it could be adopted by unanimous consent, failing which, it would have to lie until the next meeting, when a two-thirds vote would pass it. This ruling ended the discussion.

THE JOINT SESSION.

The two associations were then called to order in joint session, and President Goddard took the chair.

The consideration of the fourth subject was passed over by unanimous consent, and the next subject, which was stated as follows, was taken up:

"The Removal of Dental Pulp, and the After Treatment."

Dr. Doyle objected to passing this question without discussion. He would like to hear some one give the proper causes for the removal of dental pulp. He was requested to begin the discussion himself, and proceeded to give an instance of a case which he had treated. The patient had had the pulp of the tooth extracted, and had not returned for further treatment until some time had elapsed. Dr. Doyle then found the tooth in a bad condition, with the periosteum inflamed and pus exuding from the cavity. He would like to hear what treatment other members would have pursued in this case.

Various members participated in a humorous discussion, still feeling, perhaps, the refreshing influence of a morning clinic. When members were brought down to the consideration of the case presented by Dr. Doyle, the discussion took a wide range, embracing the consideration of dental abscess, the extraction of tooth pulp or nerve, the use of tin foil for filling, and the use of artificial dentine and various materials.

The president called the attention of the members to the question, when, after some discussion of the original question, Prof. Taft was called upon, and responded at some length, showing the difference between the healthy pus thrown off by a person of good vital powers, and the vitiated secretion, of offensive odor, and acrid, which, can never be absorbed without serious injury.

Dr. Keely introduced the subject of the use of cotton in filling, describing some cases to show the danger of its use. He strongly objected to the use of cotton in any shape for filling.

Dr. Taft had used cotton for filling, and thought it could be used without injury; if properly moistened and packed, it can afford no lodgement for secretion, and will not decompose, moistened with creosote for filling the terminal portion of root canals. Metallic filling is liable to the same objections, if not thoroughly condensed.

The sixth subject, care of children's teeth, was taken up, when Dr. J. F. Johnston, of Indianapolis, read an exhaustive essay on the subject. (This valuable paper was lost by the reporter of one of the Louisville papers.—Ed.)

After a brief discussion of this paper the joint session rose.

The Kentucky Association was again called to order, when Drs. Doyle and Wells escorted the president elect, Dr. G. W. Priest, to the place.

The president, Dr. Priest, thanked the members of the Kentucky Association, for the compliment of an election to the position of presiding officer. Nothing could make him take more interest in the welfare of the association, than he had always done, but he would endeavor to be a worthy successor to those who had held the chair.

Dr. Goddard, the retiring president, followed in an able paper; (for which, see June Register.—Ed.)

The report of the Board of Censors recommending Dr, J. F. Lambkin for membership was read and approved.

The following gentlemen were elected delegates to the American Dental Association: Drs. L. W. Wells, G. F. Baldwin, W. G. Redman and J. H. Taylor.

After adopting the report of the Finance Committee, the association adjourned.

AFTERNOON SESSION.

The State Dental Association of Kentucky, was called to order at a few minutes after three, by the president, Dr. Priest.

The following resolutions of thanks were offered by Dr. Goddard:

*Resolved*, That the thanks of the Kentucky State Dental Association, and the Indiana State Dental Association be, and they are hereby, extended to B. F. Camp, Esq., president of the School Board, for his kindness in allowing us the use of the room of the School Board for the purpose of holding our meetings.

*Resolved*, That the thanks of the Associations are hereby extended to Mr. Newman for his kindness in tendering the use of Association Hall for our meetings ; also, to the representatives of the daily papers for their full and accurate reports.

The amendment to section 1, of article 3, of the constitution, previously offered by Dr. Doyle and laid on the table until the afternoon session, was taken up and adopted unanimously. This amendment was as follows :

Any regular dental practitioner of any State other than the State of Kentucky, may be elected to honorary membership in this association upon the recommendation of the Board of Censors. And such honorary member shall be exempt from all dues and assessments ; and shall have the right to participate in all discussions, except those involving the expenditure of money. But he shall not be entitled to vote upon any question, and shall be entitled to the diploma of this association upon the payment of the usual fees charged active members; namely, $3 for graduates and $20 for non-graduates.

The amendment of Dr. Rogers in relation to the place of meeting was discussed, and, on motion, its consideration was laid over until the next annual meeting.

THE JOINT SESSION.

The Kentucky Association adjourned, and the two associations met in joint session.

Dr. Goddard moved that, as the time was short, the associations pass at once to the consideration of the tenth subject, which was stated as follows:

"What are the points necessary to be observed in the construction of Artificial Teeth ?"

The first paper offered was by Dr. J. F. Canine, entitled "Mechanical Dentistry " which was full of useful information to the profession, and an elaborate discussion of the subject in hand. (For which see another page.)

Dr. B. Oscar Doyle was called upon to read a paper upon the "Insertion of Artificial Crowns upon the Natural Root." This paper was illustrated by a specimen of pivot-tooth ready for insertion, which Dr. Doyle handed to the association to be examined. (For which see another page in this No.)

The joint session then rose, and most of the members left to attend a clinic at Dr. Seymour's office.

During the intermission the Kentucky State Dental Association was called to order, and the report of the Board of Censors, recommending that the following named gentlemen be declared honorary members of the association was adopted: Drs. J. W. Baxter, Vevay, Ind.; P. G. C. Hunt, Indianapolis; J. F. Johnston, Indianapolis; George W. Keely, Oxford, Ohio; W. F. Morrell, New Albany, Ind.

On motion of Dr. C. E. Dunn, a committee was appointed to revise the constitution, by-laws and rules, with instructions to put the amendments in their proper order.

The president appointed on this committee Drs. Doyle, Dunn and Goddard, Dr. Peabody refusing to serve on the committee on account of his engagements.

The propriety of placing none but residents of Louisville upon the committee was discussed, the conclusion reached being, that this was the only advisable course.

Dr. Doyle moved that the dentists of Louisville be allowed to change the hours of the semi-monthly meetings to suit themselves. Laid on the table until the following day.

On motion it was resolved, that when the Kentucky State Dental Association adjourn, it would adjourn to meet at the office of Dr. Priest, the next morning at half-past eight o'clock.

It was ordered that ten more diplomas be printed.

The thanks of the joint session were unanimously voted to Dr. J. F. Canine, for the hospitable entertainment which he gave the members of the association at his residence, Wednesday evening.

Dr. Merritt Wells, president of the Indiana State Dental Association, returned the thanks of the association which he represented, to the Kentucky State Dental Association for its hospitality, and invited the association to visit the Indiana Association at its annual session, on the last Tuesday in June, 1874.

Dr. W. G. Redman was elected a member of the Executive Committee, vice Dr. G. W. Priest, elected president.

With this change, the officers of the association are ;

President—Dr. G. W. Priest.

Vice President—Dr. W. M. Rogers.

Secretary—Dr. B. Oscar Doyle.

Treasurer—Dr. J. F. Canine.

Board of Censors—Drs. C. E. Dunn, F. Peabody. W. H. Goddard.

Executive Committee—Drs. C. E. Dunn, J. F. Canine, W G. Redman.

After allowing several bills for small amounts, the Kentucky State Dental Association adjourned.

